# Resistance and Inactivation Kinetics of Bacterial Strains Isolated from the Non-Chlorinated and Chlorinated Effluents of a WWTP

**DOI:** 10.3390/ijerph10083363

**Published:** 2013-08-06

**Authors:** Sylvia Martínez-Hernández, Gabriela A. Vázquez-Rodríguez, Rosa I. Beltrán-Hernández, Francisco Prieto-García, José M. Miranda-López, Carlos M. Franco-Abuín, Alejandro Álvarez-Hernández, Ulises Iturbe, Claudia Coronel-Olivares

**Affiliations:** 1 Instituto de Ciencias Básicas e Ingeniería, Área Académica de Química, Universidad Autónoma del Estado de Hidalgo, Carr, Pachuca-Tulancingo km. 4.5 s/n, Ciudad del Conocimiento, Mineral de la Reforma, Hidalgo, C.P. 42184, Mexico; E-Mails: smhjunio@gmail.com (S.M.-H.); g.a.vazquezr@gmail.com (G.A.V.-R.); icelabeltran@yahoo.com.mx (R.I.B.-H.); prietogmx@yahoo.com.mx (F.P.-G.); alvarez@uaeh.edu.mx (A.A.-H.); 2 Laboratorio de Higiene Inspección y Control de Alimentos, Dpto. de Química Analítica, Nutrición y Bromatología, Facultad de Veterinaria, Universidad de Santiago de Compostela, Lugo 27002, Spain; E-Mails: josemanuel.miranda@usc.es (J.M.M.-L.); carlos.franco@usc.es (C.M.F.-A.); 3 Instituto de Ciencias Básicas e Ingeniería, Área Académica de Biología, Universidad Autónoma del Estado de Hidalgo, Carr, Pachuca-Tulancingo km. 4.5 s/n, Ciudad del Conocimiento, Mineral de la Reforma, Hidalgo, C.P. 42184, Mexico; E-Mail: darwinianman@gmail.com

**Keywords:** bacterial resistance, inactivation response, sodium hypochlorite, wastewater treatment, disinfection, reclaimed water

## Abstract

The microbiological quality of water from a wastewater treatment plant that uses sodium hypochlorite as a disinfectant was assessed. Mesophilic aerobic bacteria were not removed efficiently. This fact allowed for the isolation of several bacterial strains from the effluents. Molecular identification indicated that the strains were related to *Aeromonas hydrophila*, *Escherichia coli* (three strains), *Enterobacter cloacae*, *Kluyvera cryocrescens* (three strains), *Kluyvera intermedia*, *Citrobacter freundii* (two strains), *Bacillus* sp. and *Enterobacter* sp. The first five strains, which were isolated from the non-chlorinated effluent, were used to test resistance to chlorine disinfection using three sets of variables: disinfectant concentration (8, 20 and 30 mg·L^−1^), contact time (0, 15 and 30 min) and water temperature (20, 25 and 30 °C). The results demonstrated that the strains have independent responses to experimental conditions and that the most efficient treatment was an 8 mg·L^−1^ dose of disinfectant at a temperature of 20 °C for 30 min. The other eight strains, which were isolated from the chlorinated effluent, were used to analyze inactivation kinetics using the disinfectant at a dose of 15 mg·L^−1^ with various retention times (0, 10, 20, 30, 60 and 90 min). The results indicated that during the inactivation process, there was no relationship between removal percentage and retention time and that the strains have no common response to the treatments.

## 1. Introduction

Reclaimed water is primarily used for agriculture and recreational activities in developing countries that have limited water supplies [1,2]. Wastewater is usually treated in activated sludge systems, which allow for the removal of high organic loads but results in the ineffective elimination of pathogens [3]. For this reason, reclaimed water may transmit human diseases and poses an environmental risk [2,4]. In wastewater treatment plants (WWTPs), secondary effluents are commonly disinfected using chemical agents, such as chlorine and its derivatives, because of their biocidal effect [5].

Sodium hypochlorite (NaClO) is a widely used disinfectant due to its strong oxidizing capacity. When it comes into contact with water, this molecule produces both HClO (hypochlorous acid, the more active fraction of chlorine) and ClO^−^ (hypochlorite ion). These fractions constitute the free available chlorine [6]. NaClO affects the plasmatic membranes of bacterial cells and disables enzymatic active sites. NaClO also diminishes the biological functions of proteins, and it produces deleterious effects on DNA. Because HClO predominates, these effects are potentiated at low pH values. This is attributed to a higher penetration of the disinfectant through the bacterial cell envelope [7,8].

This type of chemical disinfection is not always effective against pathogenic bacteria because the concentration of residual chlorine needed to inactivate each type of microbe is specific [1]. Environmental and physicochemical factors must also be considered during inactivation because they affect the efficacy of the disinfectant. Thus, it seems difficult to establish common conditions that will satisfactorily inactivate all species of microorganisms, especially for pathogens that have developed resistance to disinfectants [9,10]. 

The mechanism by which bacteria acquire resistance to chlorine and its derivatives is not well understood. It is known that environmental conditions (e.g., temperature) can diminish resistance to stress factors such as chlorine [11]. The term *stressome* describes the phenomenon of indirect resistance that occurs when additive environmental and stress factors cause the expression of genes that increase bacterial resistance [12]. Additionally, suspended solid particles and organic matter can provide protection to microorganisms by generating a demand for residual chlorine, which decreases the availability of chlorine and weakens the disinfection process. Microbial aggregation is another factor that confers resistance to chlorine disinfection [13,14].

Several waterborne diseases are caused by opportunistic and pathogenic bacteria that are found at lower levels than the traditional indicators of water quality [15,16]. The methods for the detection of these microorganisms are complex, and some species show greater resistance to high doses of disinfectants [17]. Consequently, the simple presence/absence method that is traditionally used to indicate treated wastewater quality does not guarantee the presence or absence of opportunistic and pathogenic bacteria [3]. New strategies are currently being developed to decrease the presence of pathogenic microorganisms in secondary effluents. To this end, it is important to assess the effects that different doses of disinfectants have on microbes, the retention times the microbes are exposed to, and the temperature of the milieu [4,10,18,19,20]. 

The objectives of this research were: (1) to assess the microbiological quality of water by counting mesophilic aerobic bacteria at different points of the WWTP; (2) to isolate and identify bacterial strains from the non-chlorinated and chlorinated effluents; (3) to evaluate the resistance of bacteria, isolated from the non-chlorinated effluent, to NaClO disinfection. The experimental conditions included: exposure to different doses of NaClO, to different contact times with the disinfectant and at different temperatures; and (4) to assess the response of the bacterial strains, isolated from the chlorinated effluent, before NaClO treatment by investigating the kinetics of inactivation at a single common dose using various contact times.

## 2. Materials and Methods

### 2.1. Microbiological Quality

#### 2.1.1. Sampling

Water samples were obtained from the WWTP at the Instituto Tecnológico de Estudios Superiores de Monterrey in Hidalgo, Mexico, in October 2009 and March 2010. This plant treats municipal wastewater using a conventional activated sludge process with extended aeration, and the tertiary treatment is chemical disinfection using NaClO (11%) by a dripping process. The disinfectant dose used in the WWTP is approximately 15 mg·L^−1^; this dose guarantees a residual chlorine concentration of 0.5 mg·L^−1^, as recommended by international water treatment regulations [21]. The samples were collected as follows: (1) at the influent; (2) at the discharge point of the secondary, non-chlorinated, effluent; and (3) at the discharge point of the chlorinated effluent. Temperature and pH parameters were measured *in situ* using a multiparameter water quality meter equipment (HI 8014, Hanna Instruments, Padova, Italy). All procedures were performed according to the protocols described in the Standard Methods for the Examination of Water and Wastewater [22].

#### 2.1.2. Mesophilic Aerobic Bacterial Counts

The microbiological quality of water from the WWTP was determined by calculating the mesophilic aerobic bacteria removal percentage at the three points of sampling. The quantity of colony-forming units (CFUs) was assessed using the 10-fold serial dilution method. Each dilution was plated in duplicate on inverted standard count agar (Bioxon, Queretaro, Mexico) and incubated at 37 °C for 24 h. The results of the quantifications are reported as the log_10_(CFU·100 mL^−1^) and as percentages.

### 2.2. Isolation and Identification of Bacterial Strains

#### 2.2.1. Isolation of Bacterial Strains

The plates of mesophilic aerobic bacteria were used to isolate bacterial strains randomly. Only colonies approximately 1 mm in diameter that were completely separated from each other were collected and cultured again. The resulting strains included five strains from the non-chlorinated effluent and eight strains from the chlorinated effluent. 

#### 2.2.2. Preparation of Bacterial Suspensions

A suspension was prepared for each of the thirteen strains. These cultures were inoculated in triplicate into flasks containing 200 mL of nutritional culture medium (Bioxon). The cultures were then incubated at 37 °C until the suspension was standardized to 0.5 McFarland (1.5 × 10^8^ CFU·mL^−1^), as reported by Cavalieri [23]. This reading was the initial cell density for each assay. Cell density values were obtained at 460 nm using a Genesys 10 UV-visible spectrophotometer (Thermo Scientific, West Palm Beach, FL, USA).

#### 2.2.3. Molecular Identification of Bacterial Strains

Gene amplification, sequencing and molecular identification of the thirteen bacterial strains were performed at the Universidad de Santiago de Compostela in Spain. Frozen strains were transported in media containing glycerol (20%). In the laboratory, the strains were reactivated in brain-heart infusion broth (Difco, Franklin Lakes, NJ, USA) at room temperature for 24 h. The strains were then purified and cultured on plate count agar (Liofilchem, Via Scozia, Italy) to evaluate the growth of viable bacteria. To ensure proper DNA extraction, tubes with enriched cultures were grown in duplicate in brain-heart infusion agar at 30 °C for 48 h. The extraction and purification of DNA was performed using a Qiagen extraction kit (Hilden, Germany). DNA was quantified using a Qubit fluorometer (Invitrogen, Carlsbad, CA, USA). The amplification of DNA fragments was performed using a MyCycler Thermocycler (BioRad, Hercules, CA, USA) and the universal primer pair for the 16S rRNA gene: p8FPL/p806R [24]. DNA amplicons were tested by gel electrophoresis using the SYBR safe marker (BioRad). The sequences were obtained using an automatic sequencing system (ABY 3730XL DNA Analyzer, Applied Biosystems, Foster City, CA, USA). The sequence alignments were performed using ClustalX2 6.0-2010 and Chromas Lite 2.01-2005 software. Finally, the sequences were compared to the GenBank database to assign the closest formal taxon to each sequence.

### 2.3. NaClO Resistance Tests

To assess bacterial resistance to chlorine, three treatments were tested on the five bacterial strains isolated from the non-chlorinated effluent. In treatment I, NaClO (11%) was added to generate concentrations of 8, 20 and 30 mg·L^−1^ in dilution bottles that contained 90 mL of sterilized saline solution and 10 mL of each bacterial suspension. The doses tested are similar to and higher than those recommended for municipal wastewater disinfection processes [25] carried out in a WWTP. The strains were exposed to contact times (T) of 0, 15 and 30 min at 20 °C. After these contact times, 100 µL of the solution was spread-plated in duplicate on trypticase soy agar (Dibico, Mexico City, Mexico) at 37 °C for 24 h. In treatment II, the same experimental procedure was performed on each strain, but the temperature was raised to 25 °C. Treatment III was performed under the same conditions but at a temperature of 30 °C. Finally, the CFUs were quantified using a Quebec type colony counter (Sol-Bat, Puebla, Mexico), and the results were reported as the log_10_(CFU·100 mL^−1^). The reduction of the bacterial content for each experiment was depicted graphically by the relation log_10_(N/N_0_) *vs.* log_10_(C_0_ T).

### 2.4. Inactivation Kinetics of the Bacterial Strains

The kinetics of inactivation were analyzed for the eight strains isolated from the chlorinated effluent and identified by molecular techniques. In dilution bottles, 90 mL of sterilized saline solution, 15 mg·L^−1^ of NaClO (11%), and 10 mL of each bacterial suspension were mixed. The strains were exposed to this single dose of disinfectant for contact times of 0, 10, 20, 30, 60 and 90 min at room temperature. 

After these contact times with the disinfectant, 100 µL of each solution was spread-plated in duplicate on Mueller Hinton agar (Bioxon) at 37 °C for 24 h. Subsequently, CFUs were quantified and reported as log_10_(CFU·100 mL^−1^). The inactivation of the eight bacterial strains was verified by calculating two removal percentages: the removal percentage at 90 min and the maximum removal percentage reached (at any retention time). Inactivation was also expressed as the log_10_(CFU·100 mL^−1^).

### 2.5. Statistical Analysis

The results of each NaClO resistance test (Section 2.3), including the mean and standard deviation, were calculated for the treatment response of the five bacterial strains, isolated from the secondary effluent, using Sigmaplot version 10 software (2006). 

A four-way ANOVA test was conducted to analyze the resistance of the five strains to treatments I, II and III (Section 2.3). To establish optimal values for each parameter (*i.e.*, contact time, dose of disinfectant and temperature) for removing the five strains with the highest efficiency, an analysis of variance was conducted using Advanced Systems and Designs software (version 2.5, American Supplier Institute, Santa Clara, CA, USA) to perform the Taguchi method using an orthogonal array. In all tests, data were transformed into natural logarithms.

For the kinetics of inactivation (Section 2.4), the means and standard deviations measured for each isolated strain were compared. To determine the differences between isolated bacterial strains, two- factor variance analysis with one average per group were performed using Student’s *t*-test. 

## 3. Results and Discussion

### 3.1. Microbiological Quality

#### 3.1.1. Sampling

The mean temperature values at the three points of the WWTP were: 20.39 °C at the influent, 19.9 °C at the non-chlorinated effluent and 20.35 °C at the chlorinated effluent. The average pH values were 8.66, 8.35 and 8.43 at the same points, respectively. These values are similar to those previously reported for the same WWTP [21]. These environmental conditions are suitable for the potential growth of microorganisms.

#### 3.1.2. Mesophilic Aerobic Bacterial Count

Mesophilic aerobic bacteria were quantified in the samples from the three points at the WWTP, as described in Section 2.1.2. In the first sampling, 8.9 × log_10_(CFU·100 mL^−1^) were measured in the influent, while 5.8 and 6.4 × log_10_(CFU·100 mL^−1^) were measured in the non-chlorinated and the chlorinated effluents, respectively. These results correspond to 99.91% and 99.68% microbial removal, respectively. 

For the second sampling, the results were 9.2 × log_10_(CFU·100 mL^−1^) in the influent, 6.9 × log_10_(CFU·100 mL^−1^) in the non-chlorinated effluent (99.54% removal) and 6.3 × log_10_(CFU·100 mL^−1^) in the chlorinated effluent (99.88% removal). In this case, the removal of bacterial cells in both effluents was more than two log units better than the influent value. However, in both samplings, the bacterial counts were above the range suggested by Salgot *et al.* [26] for direct reuse of a treated effluent; the suggested range is 1,000–10,000 CFU·mL^−1^, which corresponds to 5–6 × log_10_(CFU·100 mL^−1^).

The chlorine treatment in the WWTP is thus ineffective. However, higher concentrations must be avoided to prevent the formation of organochlorinated compounds [6]. Although the international standard was met, mesophilic aerobic bacteria were not efficiently removed from the chlorinated effluent of the WWTP. This inefficient removal allowed for the isolation of several strains from both the non-chlorinated and chlorinated effluents.

### 3.2. Isolation and Identification of Bacterial Strains

#### 3.2.1. Isolation of Bacterial Strains

A total of five bacterial strains were isolated from the non-chlorinated effluent and used for the resistance test. A total of eight strains were isolated from the chlorinated effluent and tested to analyze the kinetics of inactivation.

#### 3.2.2. Molecular Identification of Bacterial Strains

The genetic sequences of the bacterial strains isolated from both the non-chlorinated and chlorinated effluents of the WWTP were compared to known sequences in the GenBank database. The closest taxa are shown in Table 1. 

The low similarity (<97%) of the 16S rRNA sequences for most of the isolated strains to bacterial taxa in the GenBank database does not support the unequivocal assignment of each strain to a formal species taxon, but it has been argued that relatively high percentages of similarity are useful for the establishment of relationships at least at the genus level [27], therefore the comparisons to come are valid. Five bacterial strains were identified as very close (98–100% similar) to an equal number of taxa (*Bacillus* sp. FRC_Y9-2, *Citrobacter freundii^b^*, *Escherichia coli*, *Kluyvera cryocrescens^a^* and *Kluyvera intermedia*; superscripts indicate that more than one isolated strain is related to the same bacterial taxon). The other eight bacterial strains were less similar to the closest taxa, as shown in Table 1.

**Table 1:** Similarity of the isolated strains to the closest taxa identified in the GenBank database.

WWTP Effluent	Test	Closest taxon	Access number	% Similarity
Non-chlorinated	RT	*Aeromonas hydrophila* AN-2	AY987736.1	95
		*Enterobacter cloacae* A5-B25	AF406657.1	92
		*Escherichia coli*	CP002516.1	98
		*Escherichia coli* BL21	AM946981.2	89
		*Escherichia coli* PD3	FR715025.1	95
Chlorinated	IK	*Bacillus* sp. FRC_Y9-2	EF158823.1	100
		*Citrobacter freundii^a^*	NR_028894.1	96
		*Citrobacter freundii^b^*	FN997639.1	99
		*Enterobacter* sp. MS5	FN997607.1	88
		*Kluyvera cryocrescens^a^*	AM933754.1	98
		*Kluyvera cryocrescens^b^*	AM933754.1	94
		*Kluyvera cryocrescens^c^*	AM933754.1	95
		*Kluyvera intermedia*	NR_028802.1	99

Type of experiment performed: RT: Bacterial resistance test; IK: Bacterial inactivation kinetics. Superscripts indicate that more than one isolated strain is related to the same bacterial taxon.

The strains isolated from the non-chlorinated effluent were expected because their closely related taxa (*i.e.*, *Aeromonas hydrophila*, *E. coli* and *Enterobactercloacae*) represent bacteria that are commonly isolated from aquatic environments [28] and domestic wastewater [29]. Most of the strains isolated from the chlorinated effluent are related to common waterborne pathogens [28]. In contrast, strains of *Kluyvera* are often found in hospital sewage samples [30]. Shi *et al.* [31] reported the presence of *Citrobacter* sp. in chlorine-disinfected water and pipeline transportation systems. The presence of microorganisms from the genus *Bacillus* in a chlorinated effluent is likely a result of the high resistance of its endospores, which has been widely reported in the literature [25]. In fact, it has been observed that the endospores produced by *Bacillus subtilis* exhibit a similar level of resistance to oocyst-forming protists such as *Giardia* [32] and *Cryptosporidium* [33]. This feature allows for the use of this bacterial species as a surrogate in chlorine inactivation assays. The taxonomic differences that were observed between the bacterial strains identified in the chlorinated and non-chlorinated effluents are not attributable to any selective efficiency of the WWTP because the isolation of strains for further culture was random. However, the close relationship of the strains, isolated from the chlorinated effluent, with pathogenic taxa poses a potential sanitary risk, as reclaimed water from this plant is used to irrigate gardens and soccer fields. The fact that microorganisms other than coliforms were identified highlights the necessity for new indicators to improve the quality of reclaimed water [21,34].

### 3.3. NaClO Resistance Tests

The degree of resistance of each of the bacterial strains from the non-chlorinated effluent to the disinfection treatments was determined by quantifying the number of CFUs (log_10_(CFU 100 mL^−1^)). The highest and the lowest resistance values measured under different experimental conditions for each strain are shown in Table 2. 

**Table 2:** Resistance of the bacterial strains to disinfectant treatment.

Closest taxon	Log inactivation	Resistance (log_10_(CFU·100 mL^−^^1^))			
		Maximum	Treatment	Minimum	Treatment
*A. hydrophila*	0.84	10.43	TI 30 mg·L^−g^/15 min	11.27	TII 20 mg·L^−g^/0 min
*E. coli*	1.31	10.15	TI 8 mg·L^−g^/30 min	11.45	TIII 8 mg·L^−g^/30 min
*E. coli* PD3	1.86	10.62	TIII 30 mg·L^−g^/0 min	12.48	TI 30 mg·L^−g^/0 min
*E. coli* BL21	0.80	10.37	TI 8 mg·L^−g^/0 min	11.18	TII 20 mg·L^−g^/0 min
*E. cloacae*	0.81	10.38	TIII 30 mg·L^−g^/30 min	11.19	TIII 8 mg·L^−g^/0 min

TI = 20 °C. TII = 25 °C. TIII = 30 °C.

The strains most affected by the chlorination process were those related to *E. coli* and *E. coli* PD3, as they had the highest reduction of CFUs measured in logarithmic units, similar to results previously reported by Koivunen *et al.* [35] using a dose of 18 mg·L^−1^. Tree *et al.* [36] suggested that *E. coli* strains are more sensitive to free or combined chlorine than other water microorganisms, especially at temperatures near 15 °C. 

**Figure 1:**
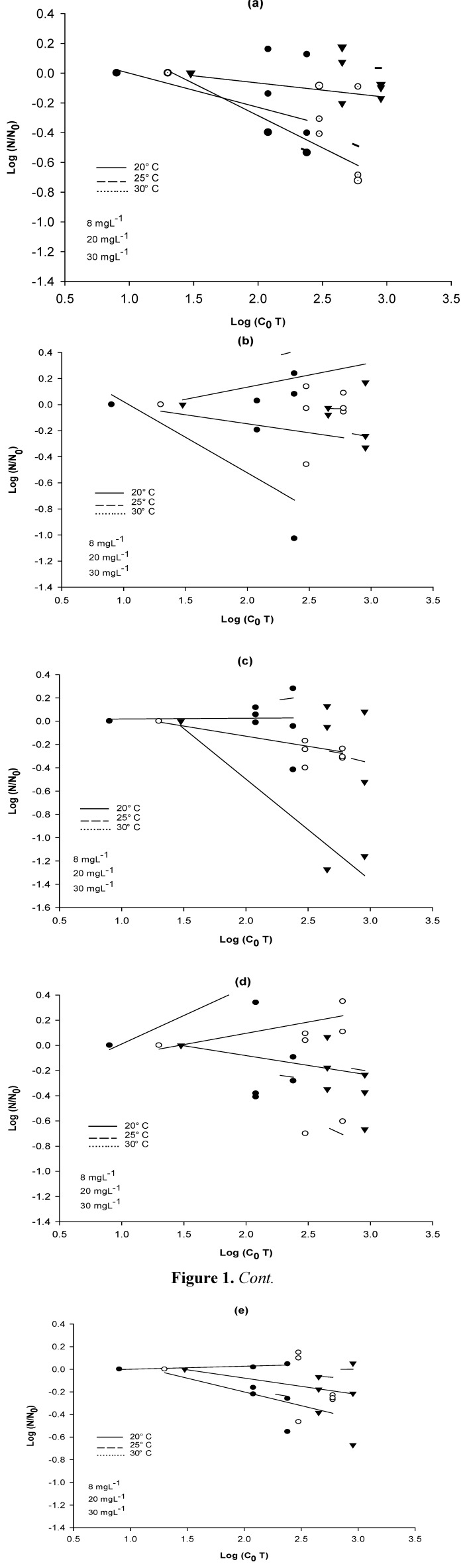
Reduction of CFUs at three temperatures (20, 25 and 30 °C) as a function of the product of the initial disinfectant concentration (mg·L^−1^) and the contact time (min). (**a**) *A. hydrophila*; (**b**) *E. coli*; (**c**) *E. coli* PD3; (**d**) *E. coli* BL21; (**e**) *E. cloacae*.

After the treatments, the remaining quantity of CFUs of each strain was correlated to the product of the initial concentration of NaClO (*C_0_*) and the retention time (*T*). Figure 1 shows the ratio of survival for each bacterial strain at the beginning (*N_0_*) and at the end (*N*) of each treatment. The results are reported as Log values. 

These results indicate that in the bacterial disinfection process there is variation in the resistance of each strain, even when using high doses of the disinfectant.

### 3.4. Inactivation Kinetics of the Bacterial Strains

The removal percentages of the bacterial strains isolated from the chlorinated effluent are shown in Table 3. Individually, the majority of the strains had a maximum removal value equal to the value observed after the maximum contact time (90 min). This result suggests that beyond a certain lethal contact time with the disinfectant, further exposure to achieve a higher inactivation response is unnecessary. The remarkable cases were the strains related to *K. cryocrescens^c^* and *K. intermedia*, which showed the highest percentage of removal after a retention time of 30 min, while at 90 min the removal percentage diminished. The strain related to *Enterobacter* sp. MS5 showed high resistance to the disinfection treatment, as a complete recovery of the initial CFU number was observed at 90 min. This unexpected result contrasts with the findings of King *et al.* [37], who achieved a 99% inactivation rate for isolated *Enterobacter agglomerans* and *E. cloacae* strains after approximately 1 min of exposure to 1 mg·L^−1^ of free residual chlorine. 

**Table 3:** Bacterial inactivation values and removal percentages obtained during analysis of the kinetics of inactivation.

Closest taxon	Removal %		Inactivation
	T = 90 min	Max%	(log_10_(CFU·100 mL^−1^))
*Bacillus* sp. FRC_Y9-2	98.87	98.87	1.96
*Citrobacter freundii^a^*	92.52	92.52	1.86
*C. freundii^b^*	99.71	99.71	2.25
*Enterobacter* sp. MS5	0	98.91 **	2.09
*Kluyvera cryocrescens^a^*	14.89	14.89	1.61
*K. cryocrescens^b^*	86.54	86.54	0.87
*K. cryocrescens^c^*	98.17	98.30 *	1.77
*K. intermedia*	65.37	87.45 *	0.9

* T = 30 min. ** T = 20 min. Superscripts indicate that more than one isolated strain is related to the same bacterial taxon.

A high inactivation response of most strains occurred with a disinfectant dose of 15 mg·L^−1^ and a variable contact time. The reduction in CFUs ranged from 0.87 to 2.25 log units. These values were similar to those reported by Macauley *et al.* [38], who observed reductions in the number of swine lagoon bacteria ranging from 2.2 to 3.4 log units with a 30 mg·L^−1^ dose.

Among the tested strains, only the strain related to *Bacillus* sp. (phylum Firmicutes) is a Gram-positive bacterium. This strain is also the only endospore-forming organism identified in this study. The particular composition of the Gram-positive bacteria cell wall, the lack of an outer membrane, a special set of genes, but more likely because the organisms had left the endospore protection, caused greater removal percentages than were observed for most of the strains, except for those related to *C. freundii^b^* and *K. cryocrescens^c^*.

Our results indicate that specific conditions are needed to eliminate each of the different bacterial species identified. These findings suggest that it is impossible to establish a single dose and a single contact time to inactivate all of the bacteria present in treated water. This finding is in agreement with Dow *et al.* [33], who attributed different inactivation responses to changes in the physical conditions of the water, such as temperature, when testing monochloramine or ozone on a single bacterial species (*i.e.*, *Bacillus subtilis*). 

### 3.5. Statistical Analysis

#### 3.5.1. NaClO Resistance Tests

A group analysis of the inactivation dynamics of the five bacterial strains isolated from the non-chlorinated effluent indicated that the degree of resistance to the disinfection process varied. This fact can be observed in the calculations presented in Table 4.

**Table 4:** Means and standard deviations (CFU·100 mL^−1^) obtained for the bacterial strains during the disinfection process.

	T (min)					
	0		15		30	
Treatment I (20 °C)						
mg·L^−1^	*x*	*δ*	*x*	*δ*	*x*	*δ*
8	1.03E+11	6.99E+10	1.0357E+11	8.244E+10	8.244E+10	6.7504E+10
20	1.363E+11	7.47E+10	6.6674E+10	3.877E+10	9.814E+10	6.196E+10
30	6.421E+11	1.32E+12	7.3978E+10	5.553E+10	7.444E+10	7.5511E+10
Treatment II (25 °C)						
8	1.01E+11	3.69E+10	9.4558E+10	6.407E+10	9.507E+10	6.2074E+10
20	1.205E+11	6.16E+10	8.4292E+10	4.971E+10	4.568E+10	1.6236E+10
30	8.99E+10	5.93E+10	8.8934E+10	5.069E+10	4.349E+10	8.326E+10
Treatment III (30 °C)						
8	1.45E+11	6.11E+10	1.2841E+11	7.769E+10	1.078E+11	1.0039E+11
20	9.814E+10	4.39E+10	9.246E+10	7.504E+10	8.758E+10	6.5128E+10
30	9.838E+10	3.76E+10	7.4647E+10	2.888E+10	4.559E+10	1.8856E+10

**Figure 2:**
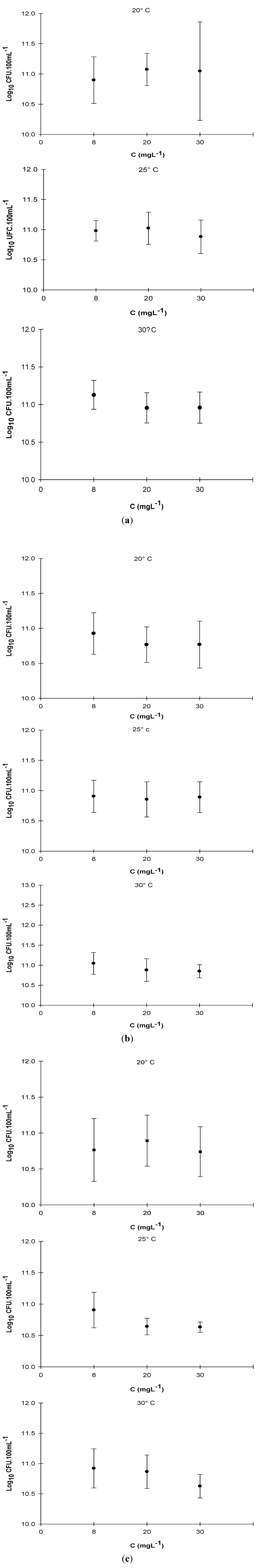
Standard deviations observed in resistance tests that used temperature and disinfectant dose as variables. C = concentration (mg·L^−1^) (**a**) T = 0 min; (**b**) T = 15 min; (**c**) T = 30 min.

The standard deviations demonstrated that the contact time with the disinfectant did not affect group resistance (Figure 2). The high standard deviations indicate the independent resistance of each bacterial strain to the treatments. This finding supports the statement at the end of Section 3.3, that it exist variation in the resistance of each strain to chlorine disinfection. This variation is likely due to a particular response to the disinfectant’s mechanism of action rather than to the disinfectant dose or to the temperature, as suggested by Cho *et al.* [19]. These results are also consistent with those reported by Berry *et al.* [12], who suggest that molecular mechanisms confer chlorine resistance to bacteria. As mentioned above, this resistance is most likely due to the expression of certain genes in response to stress factors, such as oxidizing agents, variations in temperature, osmotic shock or small amounts of organic matter present in the culture medium. Such growth conditions could presumably alter the bacterial inactivation process by reducing bacterial metabolism or by changing the permeability of the cell membrane.

**Figure 3:**
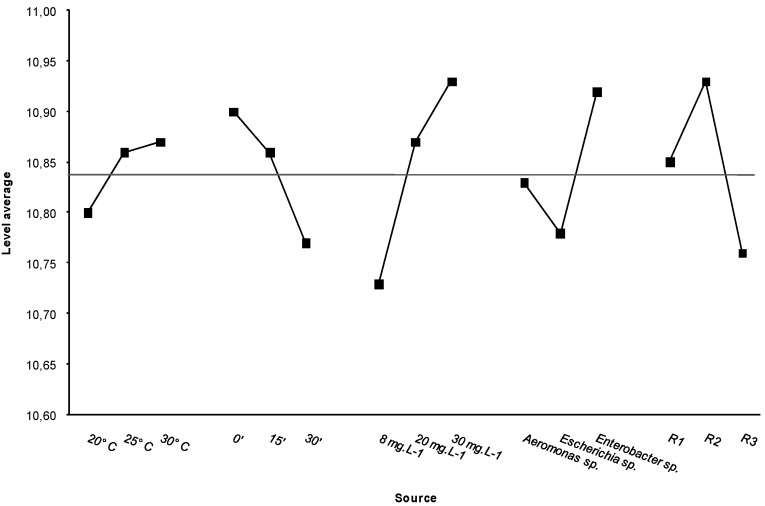
Values from the ANOVA test and the average midline of all values.

The ANOVA test showed significant differences for each set of experimental assays (Table 5). The most efficient conditions for decreasing bacterial resistance were a low temperature (20 °C), a long contact time (30 min) and a low dose of disinfectant (8 mg·L^−1^). The *E. coli* strains showed the least resistance to the treatments tested (Figure 3). These results do not support the plausibility that significant amounts of organic matter were present in the bacterial suspensions; therefore, we can dismiss the possibility that major interaction of organic matter with chlorine prevented its biocidal effect.

**Table 5:** Values from the ANOVA test.

Source	d.f.	S	V	F	ρ
A	2	0.02	0.01		
B	2	0.08	0.04	3.67	4.02
C	2	0.19	0.10	9.24	12.42
D	2	0.10	0.05	4.88	5.84
R	2	0.13	0.07	6.23	7.89
e1	16	0.86	0.05	5.16	50.23
<e>	2	0.02	0.01		19.60
TOTAL	26	1.39	0.05		100

A: temperature; B: contact time; C: concentration; D: bacterial groups; R: s/n ratio; d.f.: degrees of freedom; S: sum of squares; V: variance; F: variance ratio; ρ: percent contribution of source; e1: pooled; <e>: pooled estimate of experimental error.

#### 3.5.2. Inactivation Kinetics of the Bacterial Strains

**Figure 4:**
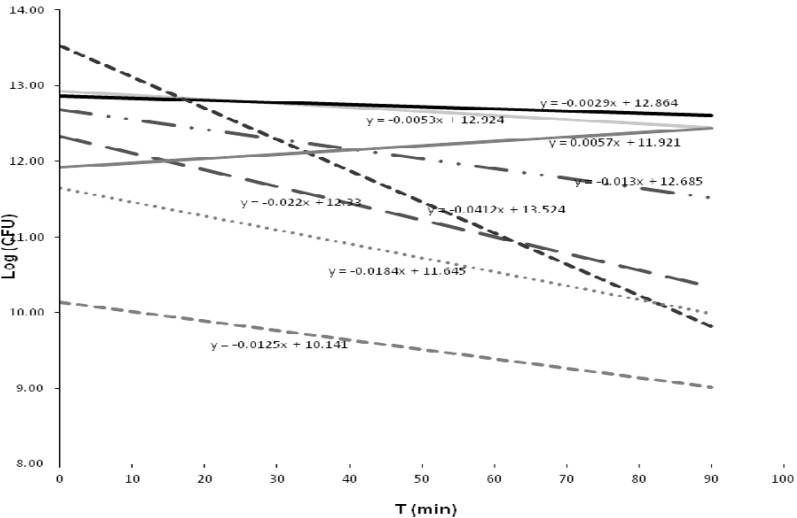
Response of the bacterial strains to the treatment, as represented by the trend line and slope value. *K. cryocrescens^a^* (*y* = −0.012*x* + 10.14). *K. cryocrescens^b^* (*y* = −0.005*x* + 12.92). * K. cryocrescens^c^* (*y* = −0.018*x* + 11.64). *K. intermedia* (*y* = −0.002*x* + 12.86). *C. freundii^a^* (y = −0.013*x* + 12.68). *C. freundii^b^* (*y* = −0.0412*x* + 13.524). *Bacillus* sp. FRC_Y9-2 (*y* = −0.022*x* + 12.33). *Enterobacter* sp. MS5 (*y* = 0.0057*x* + 11.92).

The relationship between bacterial inactivation and contact time is shown in Figure 4. The standard deviation plot for each bacterial strain demonstrated that most of the strains reacted in a different way to the disinfection process, most likely because the disinfectant had not lost its biocidal capability, although few active fractions of chlorine could have been formed [7]. Of interest were the strain related to *C. freundii^b^*, which exhibited an accelerated inactivation response, and the strain related to *Enterobacter* sp. MS5, which had the highest CFU recovery with the longest contact time. 

Similarly, the mean analysis for all the strains showed similar inactivation responses in three groups of bacteria (A, B and C), while in two other groups (D and E), the responses were independent (Table 6). There were significant differences in the inactivation tests of the last two groups when compared with the former groups.

Two-factor variance analysis found no relationships or significant differences between bacterial inactivation and contact times, particularly when the tabulated Fisher number (*F_t_* = 2.69) and the calculated Fisher number (*F_c_* = 2.53) were compared. This result was not observed for the individual inactivation trend of each strain; here again, significant differences were found (*F_c_* 2.21 > *F_t_* 2.17). This finding was also supported by an individual analysis of each strain using Student’s *t*-test.

**Table 6:** Comparison of means using Student’s *t*-test.

Group	Closest taxon	Mean	Sd	T cal	T tab
A					
	*K. cryocrescens^b^*	12.64	0.34	0.68	2.17
	*K. intermedia*	12.76	0.28		
B					
	*Bacillus* sp. FRC_Y9-2	11.56	0.87	0.15	
	*K. cryocrescens^c^*	11	0.79		
C					
	*K. cryocrescens^a^*	9.7	0.76	0.43	
	*C. freundii^a^*	12.23	0.58		
D, E					
	*C. freundii^b^*	12.53	0.89	3.34	
	*Enterobacter* sp. MS5	12.11	0.75		
Among groups					
A–B	A	12.7	0.31	3.9	
	B	11.27	0.83		
C–B					
	C	10.97	0.67	2.82	
	B	11.27	0.83		

T cal = calculated Student’s t value; T tab = tabulated Student’s t value.

However, the possible protective effect of organic matter upon the bacterial cells during chlorine disinfection must be considered. A higher chlorine demand caused by organic compounds present in the culture medium causes a rapid decline in the availability of free chlorine. In experiments performed by Virto *et al.* [14] with a calculated organic load of 1,120 ppm, the concentration of NaClO (10%) had to be raised several times to achieve bacterial inactivation. The disinfectant dose had a clear but differential effect on the bacterial strains only above 15–35 mg·L^−1^, which was in contrast to the low chlorine concentration (approximately 1 mg·L^−1^) necessary to completely inactivate the same microbial populations when tested in a distilled water milieu. In our experiments, the dose of 15 mg·L^−1^, although relatively high, efficiently achieved a significant inactivation response in each bacterial strain within the time intervals considered. Thus, it is unlikely that the free chlorine was prevented from interacting with the bacterial cells by organic matter in the experiments conducted in this study, although this possibility cannot be completely dismissed. It is also possible that morphological or physiological features of each bacterial strain contribute to chlorine resistance.

## 4. Conclusions

Our analysis demonstrated that the secondary treatment of active sludge does not efficiently remove the mesophilic aerobic bacteria from the wastewater influent of the WWTP under study. A higher removal of bacteria did not occur even after the chlorination treatment, meeting the international standard, was performed. Therefore, the disinfection treatment using only NaClO in this WWTP is ineffective; another treatment could be used in combination with chlorine to increase removal efficiency of bacteria.

Several bacterial strains were isolated from the non-chlorinated and chlorinated effluents of the WWTP. A comparison of the gene sequences of the 16S rRNA of these strains with known taxa demonstrated that a diversity of bacteria is present in municipal wastewater, most of which are different from the traditional coliform indicators.

In tests of resistance to NaClO, the standard deviations indicated that each bacterial strain responded independently when experimental conditions vary. The ANOVA test demonstrated that the most efficient conditions for decreasing the bacterial resistance of all strains were low temperature (20 °C), increased contact time (30 min) and low doses of disinfectant (8 mg·L^−1^). The strains related to *E. coli* taxa showed the least resistance to the experimental treatments. 

In the bacterial inactivation experiments, a modest reduction in log units was achieved, although there was no clear relationship between removal percentages and specific retention times. Statistical analyses indicated that each strain has a particular inactivation response. It would be useful to test different inactivation conditions on distinct groups of opportunistic and pathogenic bacterial species that are phylogenetically related to each other and to address the impact of organic matter content on the efficiency of chlorine disinfection for these groups of species.

It must be stressed that bacterial cells that remained viable after both the disinfection tests and the analysis of inactivation kinetics using NaClO (11%) are resistant. Especially, the bacterial strains isolated from the chlorinated effluent represent a serious sanitary risk because most of strains are phylogenetically related to species and genera that include opportunistic and pathogenic microorganisms. These strains are non-fecal in origin and are different from coliforms. Thus, there is an urgent need to improve reclaimed water regulations to include species other than the traditional indicators of water quality.
